# Prevalence and a LASSO-derived prediction model for screening-positive mild cognitive impairment among older adults in Wuhan

**DOI:** 10.1038/s41598-026-56222-0

**Published:** 2026-06-08

**Authors:** Dajie Chen, Cen Gao, Wencai Chen, Wenzhen Li, Xiujun Liu, Qingzhou Cheng, Yameng Feng, Rong Nie, Hongping Zhang, Hua Hu

**Affiliations:** 1 Department of Health Services and Management, College of Medicine and Health Science, Wuhan Polytechnic University, No.68 Xuefu South Road, Changqing Garden, Dongxihu District, Wuhan City, Hubei Province China; 2 Department of Psychosocial, Wuhan Mental Health Center, Wuhan, 430012 Hubei Province China; 3 Office of Psychosocial Services, Wuhan Hospital for Psychotherapy, Wuhan, 430012 Hubei Province China; 4 JC School of Public Health and Primary Care, The Chinese University of Hong Kong, Shatin, Hong Kong Special Administrative Region China

**Keywords:** Older adults, MCI screening, Machine learning, Cross-sectional study, Diseases, Health care, Medical research, Risk factors

## Abstract

This study aimed to investigate the prevalence of screening-positive mild cognitive impairment (s-MCI) and to develop a parsimonious prediction model using machine learning methods to identify high-risk older adults in Wuhan, China. A total of 2,190 community-dwelling adults aged ≥ 60 years were recruited through multistage cluster sampling from 30 residential committees in 13 districts. MCI screening was conducted with the Community Screening Instrument for Dementia (CSI-D). The Least Absolute Shrinkage and Selection Operator (LASSO) was employed to select predictive variables, and multiple machine learning classifiers were compared. Model performance was assessed using the area under the receiver operating characteristic curve (AUC) and decision curve analysis (DCA). SHapley Additive exPlanations (SHAP) was used to interpret the best-performing model. The screening-positive rate of MCI was 35.3%. The final prediction model retained four predictors: age, occupation, sleep disorders, and literacy level. XGBoost showed slightly better discrimination than logistic regression in the validation set (AUC: 0.693 vs. 0.671). Based on SHAP values, sleep disorders, advanced age, lower education level, and farmer occupation were the most important contributors to the prediction. The burden of screening-positive MCI is considerable among older adults in Wuhan. The LASSO-derived prediction model, comprising easily obtainable variables, can serve as a practical risk-stratification tool to support targeted early screening, without implying a unique etiological role for the selected predictors.

**Supplementary Information:**

The online version contains supplementary material available at 10.1038/s41598-026-56222-0.

## Introduction

With the acceleration of population aging, China’s adult population aged ≥ 60 years reached 280.04 million by the end of 2022, accounting for 19.8% of the total population^1^. A large body of research evidence suggests that the prevalence of cognitive impairment will increase with the growth of the older adult population^2,3^. Cognitive impairment is defined as deficits in one or more cognitive functions and can be categorized based on the severity of functional impairment into mild cognitive impairment (MCI) and dementia. MCI is the preclinical stage of dementia and has a high likelihood of transitioning to dementia^4^, with studies documenting that 10–15% of patients with MCI progress to dementia each year^5^.

China has the largest number of dementia patients in the world, accounting for approximately 25% of the global total^6^. Dementia imposes a heavy economic and social burden on China. Statistics indicate that the direct and indirect costs of treating dementia patients in China amount to trillions of dollars per year^2^. Furthermore, relevant studies have indicated that factors such as gender, age, and dietary habits are associated with an increased risk of mild cognitive impairment (MCI) development^7^. However, due to variations in participants, regions, and research methods, factors such as gender, smoking, alcohol consumption, and sleep disorders have not demonstrated consistent correlations^8,9^. China has been exploring the development of dementia-specific prevention and treatment services since 2020 and has carried out cognitive function screening and early intervention for older adults in several regions. Nevertheless, comprehensive investigations on the comprehensive risk factors of large-scale mild cognitive impairment are still scarce in China. To address the inconsistencies and regional differences in previous studies and explore more potential related factors, our research aims to investigate these aspects among the elderly aged 60 and above in different districts of Wuhan. Notably, there has been no relevant research conducted at this specific location before. With a relatively high survey coverage rate, our study investigates the potential related factors of Community screening positive MCI (suspected-MCI) from three aspects: demographic characteristics, behavioral lifestyles, and health status, aiming to construct a simplified, clinically applicable prediction model for s-MCI and provide a reference for the prevention of s-MCI.

## Methods

### Study design and participants

This study was based on the Basic Public Health Service Program, the Cognitive Function Screening and Early Intervention Program for Older Adults launched by the National Health Commission in 2023, and which has been implemented in hundreds of cities across China, including Wuhan. Cognitive function and health screening was performed by multistage cluster sampling in Caidian, Hanyang, Huangpi, Wuchang, and Xinzhou districts, which were randomly selected as screening areas from the 13 administrative districts of Wuhan. The five selected districts represent a mix of urban (Wuchang, Hanyang), suburban (Caidian), and rural (Xinzhou, Huangpi) areas, ensuring socioeconomic diversity. In each of these five districts, we randomly selected two subdistricts. In each subdistrict, we randomly selected two residential (village) committees. In each residential (village) committee, all permanent residents (including those with local household registrations and those who had lived in the city for over half a year) aged 60 and above (born before December 31, 1962) underwent cognitive function and health screening. The following exclusion criteria for MCI screening were adopted: (1) incomplete or questionable data; (2) severe hearing or vision loss preventing the completion of the cognitive assessment; and (3) confirmed diagnosis of dementia. A total of 2,402 participants from 30 residential (village) committees completed the survey. After excluding survey data with unclear primary records and incomplete information, A total of 2,190 valid participants were included.

### Ethics approval and consent to participate

All methods were carried out in accordance with the Declaration of Helsinki and regulations of the National Health Commission of China. The study was approved by the Ethical Review Committee of Wuhan Mental Health Center (approval No. KY2023.0412.03). Before the survey began, written informed consent was obtained from all participants and/or their legal guardians.

### Screening procedures

All surveys were completed one-on-one with participants in a quiet environment through home visits by staff from subdistrict community health centers who received uniform training. Participants were first asked to fill out a Basic Information Questionnaire, which included demographic characteristics (gender, age, marital status, occupation, literacy level, monthly income, household size), behavioral lifestyle (smoking, alcohol consumption, exercise days/week), and health status (body mass index (BMI), number of chronic diseases, depression, anxiety, sleep disorders). This was followed by the Resident Mental Health Questionnaire, which used the Patient Health Questionnaire-9 (PHQ-9), Generalized Anxiety Disorder Questionnaire-7 (GAD-7), and Insomnia Severity Index (ISI) scale to assess the mental health and sleep quality of older adults. The psychiatrists then reviewed the diagnoses of participants who screened positive for depression/anxiety and 15% who screened negative. Finally, to assess their cognitive function, participants completed the Community Screening Instrument for Dementia (CSI-D), and the psychiatrists reviewed the diagnoses of those who screened positive for MCI and 15% who screened negative.

### Positive screening criteria

Assessment of cognitive function: Researchers from various countries have developed numerous dementia screening scales applicable to different countries and groups. Among which, the CSI-D has displayed good reliability and validity when applied to the Chinese population and has been widely used in China. A total CSI-D score of ≤ 7 indicates s-MCI, while a score of > 7 indicates no cognitive impairment. Assessment of depression: A PHQ-9 score > 4 indicates symptoms of depression. Assessment of anxiety: A GAD-7 score of > 4 indicates symptoms of anxiety. Assessment of insomnia: An ISI score of ≥ 8 indicates clinically significant insomnia. It is important to note that the CSI-D is a screening tool and not a diagnostic instrument; therefore, the term ‘MCI’ in this study refers to screening-positive cases, which may include false positives and do not equate to a clinical diagnosis.

### Model development and comparison

First, R software (glmnet4.1.2) was used to conduct the least absolute shrinkage and selection operator (LASSO) regression analysis and adjust the variable screening and complexity. Subsequently, the screened features were used to develop classifiable models. Five ML models namely K Nearest Neighbours (KNN), Logistic Regression (LR), Random Forest (RF), Support Vector Machine (SVM), and eXtreme Gradient Boosting (XGboost) were used for classifying the occurrence of s-MCI. Several commonly used evaluation indexes, such as AUC, sensitivity, specificity, PPV, negative predictive value(NPV), accuracy, and F1 score, were used to evaluate the reliability of these models. Furthermore, a DCA was carried out to evaluate the utility of the decision models by quantifying the net benefit across different threshold probabilities. A binary logistic regression model was applied to analyze the associated factors for s-MCI, in which the dependent variable was the presence of MCI (yes/no),

The dataset was randomly split into training (70%) and validation (30%) sets, stratified by the outcome. To address class imbalance, the Synthetic Minority Over-sampling Technique (SMOTE) was applied to the training set. Ten-fold cross-validation was used for hyperparameter tuning. For XGBoost, we performed grid search over max_depth (3, 5, 7), learning_rate (0.01, 0.1, 0.2), and n_estimators (100, 200). The final model was evaluated on the held-out validation set. We also built logistic regression, random forest, SVM, and KNN models for comparison. For categorical variables with more than two levels, dummy variables were created with the following reference categories: literacy level (illiterate), occupation (technical personnel), and age group (60–64 years).

### Model explanation

The SHapley Additive exPlanations (SHAP) method offered global and local explanations for the model explanation. The global explanation could give consistent and accurate attribution values for each feature within a model to show the associations between input features and s-MCI. The local explanation could demonstrate a specific classification for individual patients by inputting the specific data. SHAP was used to interpret the XGBoost model. SHAP values represent the contribution of each feature to the model’s classification for an individual; they do not imply causality.

### Statistical analysis

Statistical analyses were performed using SPSS 27.0 (IBM Corp.). and R (R version 4.5.1). Continuous variables were expressed as mean ± standard deviation (SD), and categorical variables were expressed as frequency (percentage). To examine multicollinearity among candidate variables, we calculated pairwise associations using Cramér’s V for categorical variables and Pearson’s correlation for continuous variables. A correlation heatmap was generated to visualize the strength of associations (Supplementary Fig. S1). Statistical inference for categorical variables was performed using the χ^2^ test. The classified power was evaluated using the AUC, with the optimal cut-off value determined by maximizing the Youden index (sensitivity + specificity-1). All tests were two-sided, with significance set at *p* < 0.05.

## Results

### General demographics of the screening population and distribution of s-MCI detected

The 2,190 participants included 821 males (60–95 years; mean age: 69.75 ± 6.05 years) and 1,369 females (60–95 years; mean age: 69.35 ± 6.75 years). Among the total screening population, 1,417 participants had normal cognitive function, and 773 (screening-positive detection rate: 35.3%) screened positive for MCI, including 486 women (screening-positive detection rate: 35.5%) and 287 men (screening-positive detection rate: 35.0%).

Except for gender (χ^2^ = 0.051, *p* = 0.822), between-group differences in the detection rate of s-MCI were observed for all other demographic characteristic groups (all groups *p* < 0.05). The detection rate tended to increase with age, and older individuals had a higher detection rate (χ^2^ = 46.567, *p* < 0.001). Widowed older adults (χ^2^ = 28.561, *p* < 0.001) and farmers (χ^2^ = 55.569, *p* < 0.001) had the highest detection rate (47.4% and 42.7%, respectively). The detection rate tended to decrease with increasing literacy (χ^2^ = 111.559, *p* < 0.001) and monthly income (χ^2^ = 85.264, *p* < 0.001). In addition, a significant difference was observed in the detection rate among households with different sizes (χ^2^ = 10.688, *p* = 0.014). The distribution of positive MCI screening results among groups with different demographic characteristics is presented in Table 1.

**Table 1:** Demographics of the screening population and distribution of s-MCI detected.

Demographic characteristics	N (%)	s-MCI (%)	*p* value
Total population	2190	773 (35.3)	
*Gender*			0.822
Female	1370(62.56)	486(35.47)	
Male	820(37.44)	287(35.00)	
*Age groups (years)*			< 0.001
60 ~ 64	560(25.57)	167(29.82)	
65 ~ 69	696(31.78)	211(30.32)	
70 ~ 74	468(21.37)	173(36.97)	
≥ 75	466(21.28)	222(47.64)	
*Marital status*			
Single	30(1.37)	11(36.67)	< 0.001
Married	1758(80.27)	576(32.76)	
Separated/Divorced	35(1.60)	12(34.29)	
Widowed	367(16.76)	174(47.41)	
*Occupation*			< 0.001
Technical personnel	392(17.90)	99(25.26)	
Retirement	427(19.50)	115(26.93)	
Farmer	967(44.15)	413(42.71)	
Business/service personnel	150(6.85)	48(32.00)	
Others	254(11.60)	98(38.58)	
*Literacy level*			< 0.001
Illiterate	530(24.20)	266(50.19)	
Elementary	547(24.98)	226(41.32)	
Middle school	591(26.99)	161(27.24)	
High school and above	522(23.83)	120(22.99)	
*Monthly income*			< 0.001
0–1499 RMB	1196(54.61)	521(43.56)	
1500–2999 RMB	326(14.89)	99(30.37)	
3000–4999 RMB	541(24.70)	129(23.84)	
≥ 5000 RMB	127(5.80)	24(18.90)	
*Family size*			0.014
1	298(13.60)	127(42.62)	
2	881(40.23)	285(32.35)	
3 ~ 7	941(42.97)	334(35.49)	
8	70(3.20)	27(38.57)	
*Smoking*			0.012
Yes	459(20.96)	185(40.31)	
No	1731(79.04)	588(33.97)	
*Drinking*			0.301
Yes	344(15.71)	113(32.85)	
No	1846(84.29)	660(35.75)	
*Exercise days/week*			0.008
< 3	937(42.79)	365(38.95)	
3 ~ 5	106(4.84)	35(33.02)	
> 5	1147(52.37)	373(32.52)	
*BMI*			0.185
Underweight	126(5.75)	52(41.27)	
Normal	1211(55.30)	440(36.33)	
Overweight	628(28.68)	204(32.48)	
Obesity	225(10.27)	77(34.22)	
*Number of chronic diseases*			0.001
0	718(32.79)	218(30.36)	
1	800(36.53)	284(35.5)	
≥ 2	672(30.68)	271(40.33)	
*Depression*			< 0.001
Yes	170(7.76)	81(47.65)	
No	2020(92.24)	692(34.26)	
*Anxiety*			0.195
Yes	136(6.21)	55(40.44)	
No	2054(93.79)	718(34.96)	
*Sleep disorders*			< 0.001
Yes	496(22.65)	219(44.15)	
No	1694(77.35)	554(32.7)	

Among this screening population, 459 (21.96%) were smokers, and 344 (15.71%) consumed alcohol. A total of 937 (42.79%) participants exercised < 3 days per week, 106 (4.84%) exercised 3–5 days per week, and 1147 (52.37%) exercised > 5 days per week.As indicated in Table 1, the detection rate of s-MCI was higher among smokers than non-smokers (χ^2^ = 6.378, *p* = 0.012). Alcohol consumption had no significant effect on the detection of s-MCI in older adults (*p* > 0.05). The detection rate among older adults tended to decrease with increasing exercise days/week, and between-group differences in the detection rate were observed (χ^2^ = 9.602, *p* = 0.008).

Among this screening population, 126 (5.75%) were underweight, 1,211 (55.30%) had a normal BMI, 628 (28.68%) were overweight, and 225 (10.27%) were obese. In addition, 718 (32.78%) participants did not suffer from chronic diseases, 800 (36.53%) from one chronic disease, and 672 (30.68%) from two or more chronic diseases. The detection rates of depression, anxiety, and sleep disorders were 7.76%, 6.21%, and 22.65%, respectively.

As indicated in Table 1, the detection rate of s-MCI among older adults increased as the number of chronic diseases increased. The detection rate was higher among older adults with depressive symptoms than among those without such symptoms (χ^2^ = 12.309, *p* < 0.001). The detection rate was also higher among individuals with sleep disorders than those without (χ^2^ = 22.023, *p* < 0.001). No significant difference was observed in the detection rate among participants with different health status in terms of BMI (*p* > 0.05) and anxiety (*p* > 0.05).

### Factor selection for the classified model

**Fig. 1:**
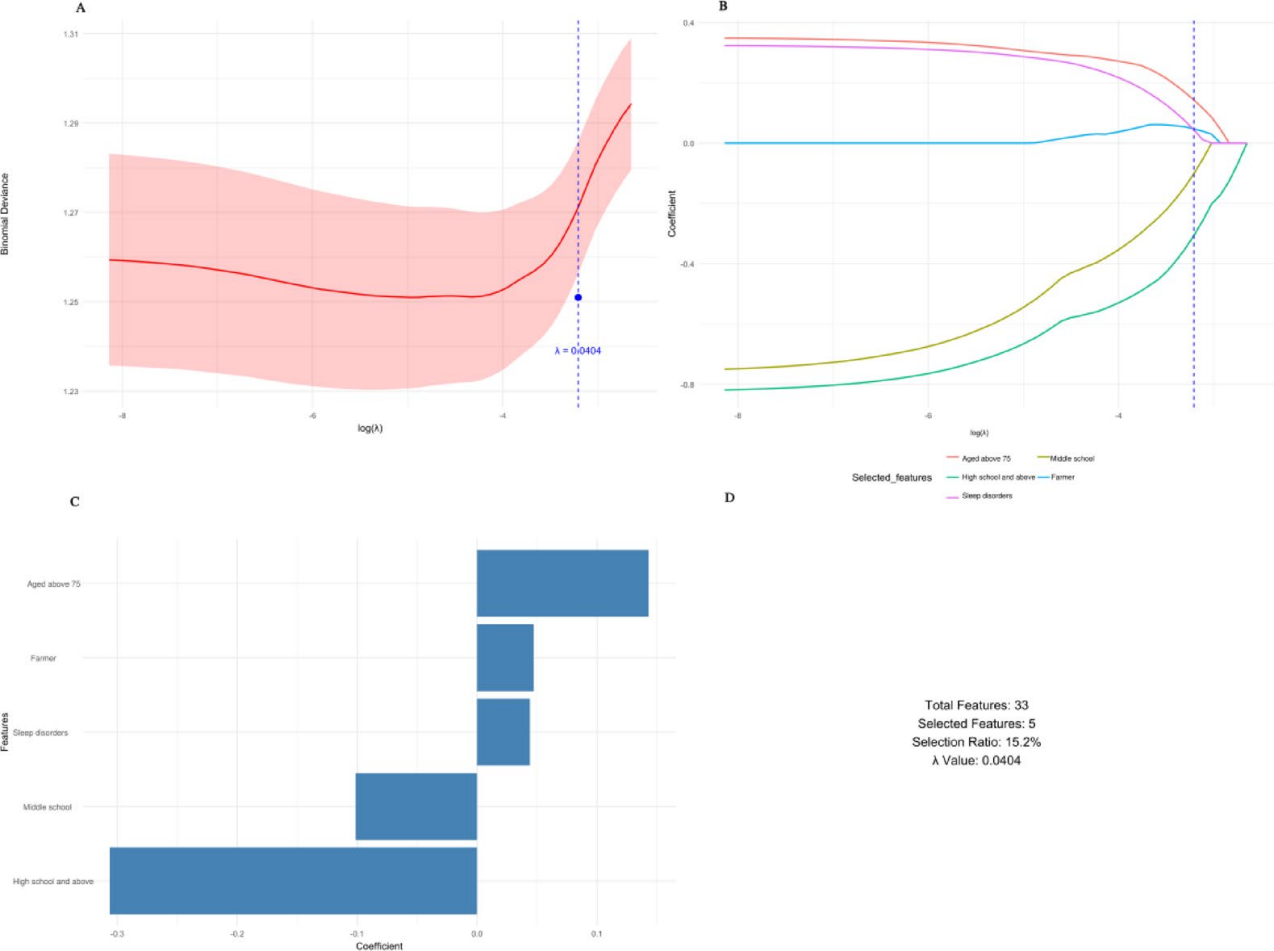
Results of the LASSO regression analysis. (**A**) Tuning parameter (λ) selection cross-validation error curve; (**B**) Plot of the LASSO coefficient profiles. (**C**) LASSO selected feature coefficients; (**D**) Feature selection statistics.

LASSO regression analysis was conducted on the remaining independent variables, with s-MCI serving as the dependent variable (Fig. 1). To increase the interpretability of the model and mitigate the risk of over fitting, The results showed (lambda = 0.0404, The maximum lambda within one standard error of the minimum error), LASSO regression reduced 33 variables to 5: age ≥ 75 years, farmer occupation, sleep disorders, middle school education, and high school education or above.

### Correlation among candidate variables

Pairwise correlation analysis revealed that the variables excluded by LASSO (income, number of chronic diseases, depression, anxiety, BMI, smoking, drinking, exercise, family size, marital status) showed strong to moderate correlations with the five variables retained in the final model. For example, Depression showed a strong association with sleep disorders (Cramér’s V = 0.41, *p* < 0.001); Number of chronic diseases was moderately correlated with sleep disorders (V = 0.21, *p* < 0.001); Income was strongly associated with literacy level (V = 0.37, *p* < 0.001) and occupation (V = 0.39, *p* < 0.001). A complete correlation heatmap is provided in Supplementary Fig. S1. These results indicate that the excluded variables did not provide independent predictive information beyond the retained set, consistent with the LASSO selection.

### The best model building and external evaluation

**Fig. 2:**
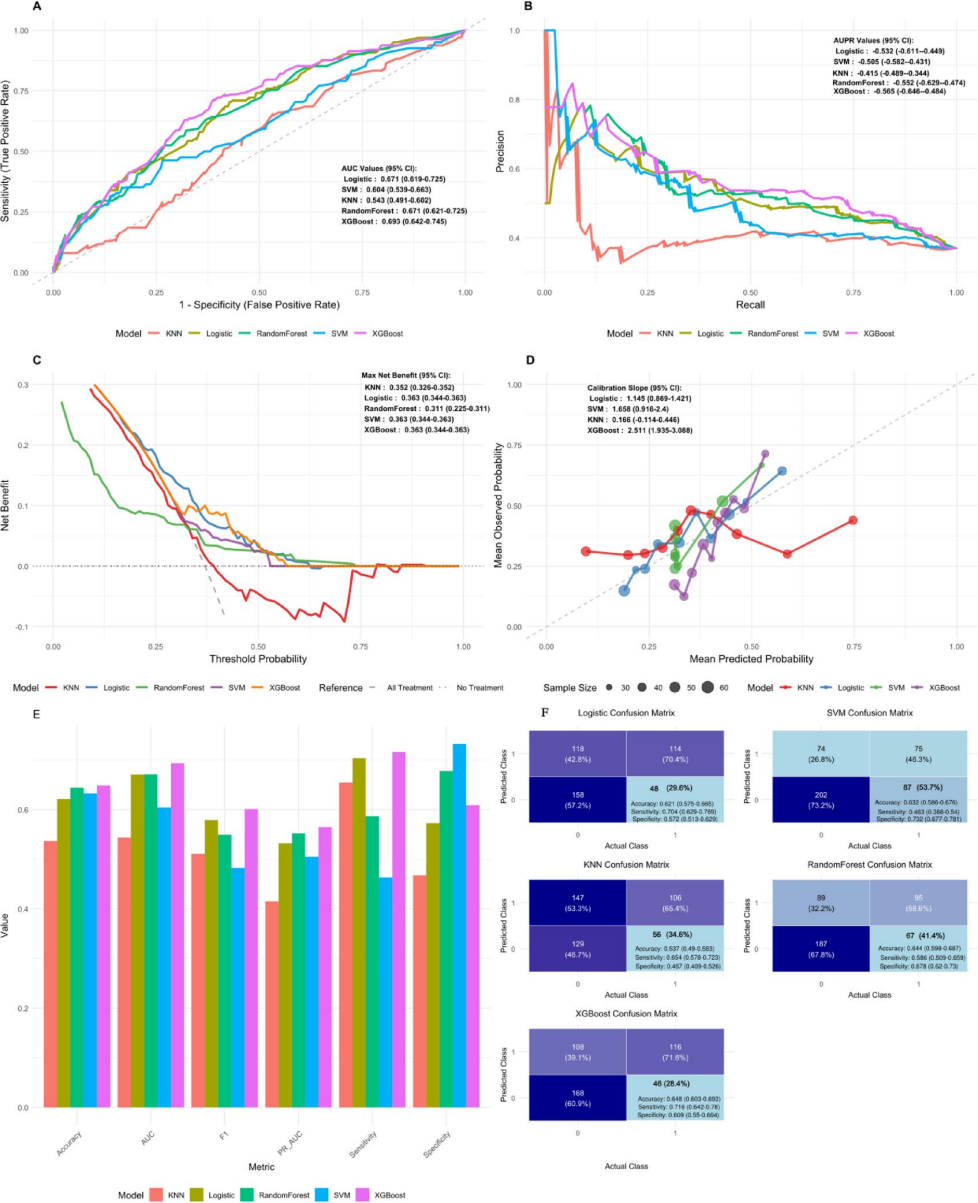
Machine learning model performance comparison report. (**A**) ROC curve comparison. (**B**) PR curve comparison; (**C**) Decision curve analysis; (**D**) Calibration curve; (**E**) Model performance metrics comparison; (**F**) Confusion matrix.

Comprehensive analysis of classified multi-model XGBoost, LR, RF, SVM, and KNN were trained and repeated 10 times. The models were evaluated using AUC values, which showed that XGBoost were highest in the validation set (Fig. 2A). The AUC indicator focuses on the classified accuracy of the model and does not tell whether the model is clinically usable or which one of the two is preferable. Therefore, the DCA, calibration curves, and PR curves were analyzed. (Fig. 2B–F). Taken together, these findings suggest that the XGBoost model was the most desirable model for this study.

**Fig. 3:**
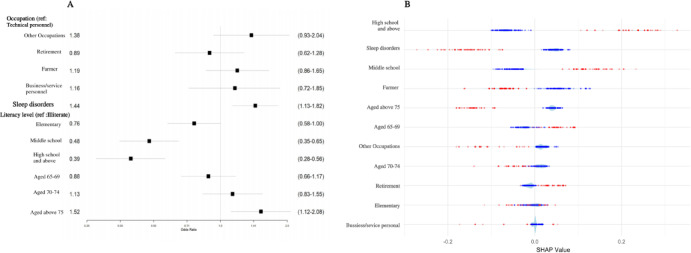
The effects of the selected variables and model interpretation. (**A**) Forest plot from logistic regression analysis showing adjusted odds ratios and 95% confidence intervals for factors associated with screening-positive MCI. (**B**) SHAP summary plot for the XGBoost model. Each point represents a Shapley value for a feature and an individual; red indicates high feature values, blue indicates low feature values. Positive SHAP values increase the classified probability of MCI. Dummy coding: literacy (reference: illiterate), occupation (reference: technical personnel), age (reference: 60–64 years).

The XGBoost model achieved an AUC of 0.693 (95% CI 0.642–0.745) on the validation set, compared with 0.671 (0.619–0.725) for logistic regression. DCA showed similar net benefit across models (Fig. 2). Given the modest difference and the value of effect size estimation, we present logistic regression results for interpretability (Fig. 3A). The results demonstrated that sleep disorders and age over 75 years were predictors to the occurrence of s-MCI. with OR values of 1.44 (95% CI 1.13–1.82) and 1.52 (95% CI 1.12–2.08), respectively. Higher literacy level is a useful predictor for s-MCI, with odds ratios of 0.48 for middle school and 0.39 for high school and above.

### Model explanation

To visually elucidate the selected variables, SHAP was utilized to illustrate how these variables classify the occurrence of s-MCI in the XGBoost model (Fig. 3B). Figure 3B depicts the four most important features (sleep disorders, age, occupation, and literacy level) in our model, the latter three are categorical variables with multiple levels and were coded as dummy variables in the model. Each feature significance line showcases all patient attributions for the outcome, represented by differently colored dots: Literacy level and sleep disorders were the most influential features. Positive SHAP values (red) for sleep disorders and age ≥ 75 indicate they were positive predictors, while higher literacy (blue) were negative factor. Figure 3B also displays the ranking of the five predictors assessed by mean absolute SHAP value, with the x-axis SHAP value indicating the importance of the classified model. Therefore, in the classified model, literacy level and sleep disorders represent the most critical factors, whereas advanced age and occupation serve as important auxiliary variables.

### Sensitivity analysis with a clinically driven full Model

To evaluate whether clinically important variables omitted by LASSO would influence model performance and to provide an explanatory complement to the predictive model, a sensitivity analysis was conducted. A fully adjusted multivariable logistic regression model was constructed, in which depression, chronic disease, income, and other a priori clinically relevant covariates were forcefully entered alongside the LASSO-selected predictors. As shown in Supplementary Fig. S2 and Supplementary Fig. S3, the clinical full model demonstrated a discriminative performance comparable to that of the LASSO-derived logistic model (AUC = 0.679; 95% CI: 0.628–0.730 vs. AUC = 0.671; 95% CI: 0.619–0.722), supporting the robustness of the parsimonious model for prediction. Notably, in this fully adjusted model, high income (≥ 5000 RMB) remained significantly associated with lower odds of s-MCI (OR = 0.50, 95% CI 0.27–0.90). This finding confirms that income carries explanatory value that should not be overlooked, even though it was not retained by LASSO for predictive parsimony.

## Discussion

To the best of our knowledge, this study is the first to analyze the screening-positive rate of s-MCI among individuals aged 60 and above in Wuhan, and to construct a simplified, clinically applicable prediction model for s-MCI and provide a reference for the prevention of s-MCI among older adults from three aspects: demographic characteristics, behavioral lifestyles, and health status.

Our findings revealed that the screening-positive detection rate of s-MCI among Wuhan residents was 35.3%, which was relatively high in China. It is crucial to emphasize that the 35.3% figure represents the proportion of participants who screened positive on the CSI-D, not a clinically confirmed prevalence. Direct comparisons with studies that used comprehensive neuropsychological assessments and clinical criteria should be made with caution. A meta-analysis published in 2021 that included 41 observational studies documented that the prevalence of s-MCI among individuals in Chinese urban communities was 12.2% (95% CI 10.6–14.2%)^10^. In 2020, a large cross-sectional study involving 12 provinces in China established that the overall prevalence of MCI among Chinese adults aged 60 years and older was 15.54% (95% CI 15.21–15.88)^11^. Although the prevalence of MCI varies across different countries and regions, ranging from 4.3% to 39.1%^12,13^, that among residents of Wuhan is still relatively high when compared with international data. For instance, a meta-analysis published in 2022 that included 66 articles involving 242,804 participants determined that the overall global prevalence of MCI among community-dwelling individuals aged 50 years and older was 15.56% (95% CI 13.24–18.03%)^14^. The significant differences in MCI detection rates across various countries and regions may be attributed not only to the inherent specificity in MCI risk that truly exists among different regions and ethnic groups, but also to the use of different screening tools in different studies (such as the Mini-Mental State Examination (MMSE), Montreal Cognitive Assessment (MoCA), Ascertain Dementia 8 (AD8), and CSI-D, etc.). Since MCI is an early stage of dementia, to shift the focus of prevention and treatment forward is critical. Moreover, given the high detection rate of s-MCI in Wuhan, analyzing the associated factors of s-MCI and emphasizing prevention on interventions targeting these factors is of considerable significance so as to achieve the prevention and control of s-MCI in Wuhan.

Our findings revealed four predictors for s-MCI, age, occupation, sleep disorders and literacy level, with advanced age being a non-modifiable associated factor. Older age was associated with a higher risk of MCI, and this finding has been confirmed in many studies^15,16^. In a cohort study with a 12-year follow-up^17^, the effect of age on cognitive impairment was associated with gray matter in the medial prefrontal cortex of the brain. The brain tissue in this region gradually atrophied with increasing age, which led to a significant decline in brain function^18^, and a particularly pronounced decline in neurological function. In addition, advanced age is a risk factor for chronic diseases such as hypertension and diabetes mellitus^19^, which can jeopardize the vascular and nervous systems, thus leading to cognitive decline in older adults^20^.

More importantly, this study identified three modifiable factors: literacy level, occupation and sleep disorders. Literacy and occupation are the main indicators of socioeconomic status, and individuals with higher levels of these displayed a lower risk of MCI, which is consistent with the results of numerous studies^11,21,22^. According to cognitive reserve theory, a higher socioeconomic status can increase the brain’s cognitive reserve by increasing cognitive stimulation for cortical development, thereby rendering the individual less susceptible to evident cognitive decline^23^; these regular and sustained stimuli also play a role in preventing the loss of neuronal connectivity and/or strengthening neuronal associations, which can compensate for neuropathological impairments related to aging, thus preventing or slowing the development of MCI^24,25^. Health effects caused by socioeconomic inequalities have always existed^26^, and have a cumulative effect, which is more prominent in old age. Early studies determined that socioeconomic factors are determinants of quality of life among older adults^27^; Low socioeconomic status may exacerbate these effects by limiting access to healthcare and increasing chronic stress, which in turn affects neuroendocrine function^27^. Therefore, relevant government departments should pay more attention to older adults with low literacy and income levels, with a special emphasis on livelihood and healthcare security. Sleep disorders may contribute to cognitive decline through multiple pathways, including activation of inflammatory processes, impaired glymphatic clearance of neurotoxic proteins, and disruption of circadian rhythms^28^.

Several methodological limitations should be noted. First, the cross-sectional design precludes causal inference; all identified factors are correlates rather than predictors in a temporal sense. Second, the use of CSI-D for screening introduces potential misclassification. Although we attempted to minimize this by using validated cutoffs, some participants may be misclassified. Such misclassification is likely non-differential with respect to predictors, potentially biasing effect estimates toward the null. Future studies should incorporate a two-phase design with diagnostic confirmation. Third, our models were validated only internally; external validation in independent populations is needed to assess generalizability. Finally, the modest improvement of machine learning over logistic regression suggests that, with a limited set of conventional predictors, simpler models may suffice. Richer data (e.g., biomarkers, neuroimaging) could better leverage complex algorithms.

Notably, the exclusion of a variable by LASSO does not imply it lacks clinical or etiological importance. LASSO selects a representative variable from a group of correlated predictors to avoid multicollinearity; therefore, the predictive information of income, depression and chronic diseases may have been partially captured by other retained variables, while their independent contribution to prediction was limited in this dataset.

Despite these limitations, our findings have practical implications. The high screening-positive rate underscores the need for targeted cognitive screening in community settings. Priority should be given to older adults with advanced age, low literacy, and sleep disorders. Early identification can facilitate timely interventions and potentially delay progression to dementia.

## Conclusion

The screening-positive rate for MCI among adults aged 60 years and above in Wuhan is relatively high. Health authorities should integrate MCI screening into routine geriatric care, focusing on individuals with advanced age, low literacy, farmer occupation, and sleep disorders. Early detection and lifestyle interventions may help mitigate the burden of cognitive decline.

## Supplementary Information

Below is the link to the electronic supplementary material.

Supplementary file1 (DOCX 415 KB)

## Data Availability

The datasets generated and/or analyzed during the current study are not publicly available due to privacy and confidentiality concerns regarding sensitive personal information, but are available from the corresponding author on reasonable request.

## Abbreviations

MCI: Mild cognitive impairment
CSI-D: Community screening instrument for dementia
BMI: Body mass index
PHQ-9: Patient health questionnaire-9
GAD-7: Generalized anxiety disorder questionnaire-7
ISI: Insomnia severity index
