# Supplementary material for: Prevalence and a LASSO-derived prediction model for screening-positive mild cognitive impairment among older adults in Wuhan
Source: Sci Rep. 2026 Jun 8;16:25998. doi: 10.1038/s41598-026-56222-0 (PMC13487169; doi:10.1038/s41598-026-56222-0)
Supplement: Supplementary file 1 [file 41598_2026_56222_MOESM1_ESM.docx]

**Supplementary Figure S1. Correlation heatmap of candidate variables.**

The color scale is as follows: darker blue represents higher correlation, while lighter colors represent weaker correlations. The diagonal (each variable with itself) is set to 1. Only variable pairs with statistically significant associations (p < 0.05) show the specific Cramér’s V value inside the corresponding cell.

**Supplementary Figure S2:** Forest plot of the fully adjusted multivariable logistic regression model for screening-positive MCI.

**Supplementary Figure S3:** Comparison of receiver operating characteristic (ROC) curves between the LASSO-selected logistic regression model and the clinical full model in the validation set.
